# Crystal structure of caesium di­hydrogen citrate from laboratory X-ray powder diffraction data and DFT comparison

**DOI:** 10.1107/S2056989017000135

**Published:** 2017-01-10

**Authors:** Alagappa Rammohan, James A. Kaduk

**Affiliations:** a Atlantic International University, Honolulu HI, USA; b Illinois Institute of Technology, Chicago IL, USA

**Keywords:** powder diffraction, crystal structure, density functional theory, citrate, caesium

## Abstract

The crystal structure of caesium di­hydrogen citrate, Cs^+^·H_2_C_6_H_5_O_7_^−^, has been solved and refined using laboratory X-ray powder diffraction data, and optimized using density functional techniques. The coordination polyhedra of the nine-coordinate Cs^+^ cations share edges to form chains along the *a*-axis. These chains are linked by corners along the *c*-axis. The un-ionized carb­oxy­lic acid groups form two different types of hydrogen bonds; one forms a helical chain along the *c*-axis, and the other is discrete. The hy­droxy group participates in both intra- and inter­molecular hydrogen bonds.

## Abstract 2

The crystal structure of caesium di­hydrogen citrate has been solved and refined using laboratory X-ray powder diffraction data, and optimized using density functional techniques.

## Chemical context   

In the course of a systematic study of the crystal structures of Group 1 (alkali metal) citrate salts to understand the anion’s conformational flexibility, ionization, coordination tendencies, and hydrogen bonding, we have determined several new crystal structures. Most of the new structures were solved using powder diffraction data (laboratory and/or synchrotron), but single crystals were used where available. The general trends and conclusions about the 16 new compounds and 12 previously characterized structures are being reported separately (Rammohan & Kaduk, 2017*a* ▸). Ten of the new structures – NaKHC_6_H_5_O_7_, NaK_2_C_6_H_5_O_7_, Na_3_C_6_H_5_O_7_, NaH_2_C_6_H_5_O_7_, Na_2_HC_6_H_5_O_7_, K_3_C_6_H_5_O_7_, Rb_2_HC_6_H_5_O_7_, Rb_3_C_6_H_5_O_7_(H_2_O), Rb_3_C_6_H_5_O_7_, and Na_5_H(C_6_H_5_O_7_)_2_ – have been published recently (Rammohan & Kaduk, 2016*a* ▸,*b* ▸,*c* ▸,*d* ▸,*e* ▸, 2017*b* ▸,*c* ▸,*d* ▸,*e* ▸; Rammohan *et al.*, 2016 ▸), and two additional structures – KH_2_C_6_H_5_O_7_ and KH_2_C_6_H_5_O_7_(H_2_O)_2_ – have been communicated to the CSD (Kaduk & Stern, 2016*a* ▸,*b* ▸).
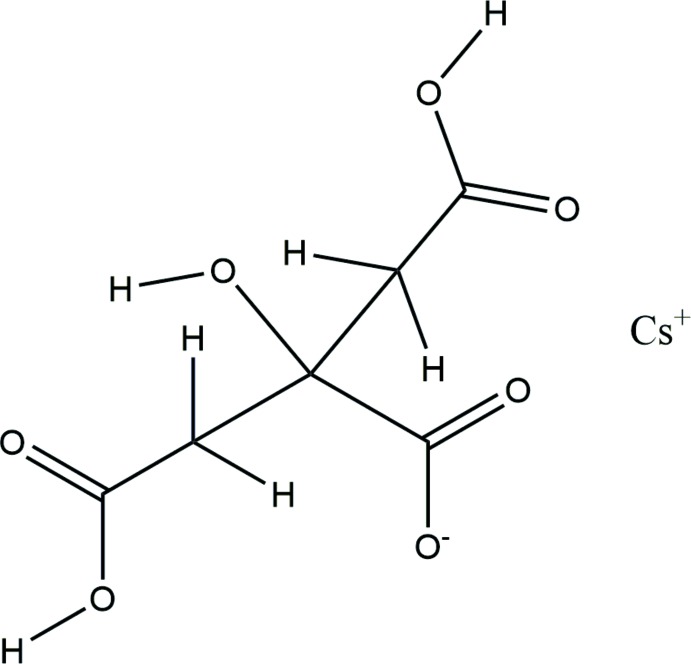


## Structural commentary   

**Figure 1:**
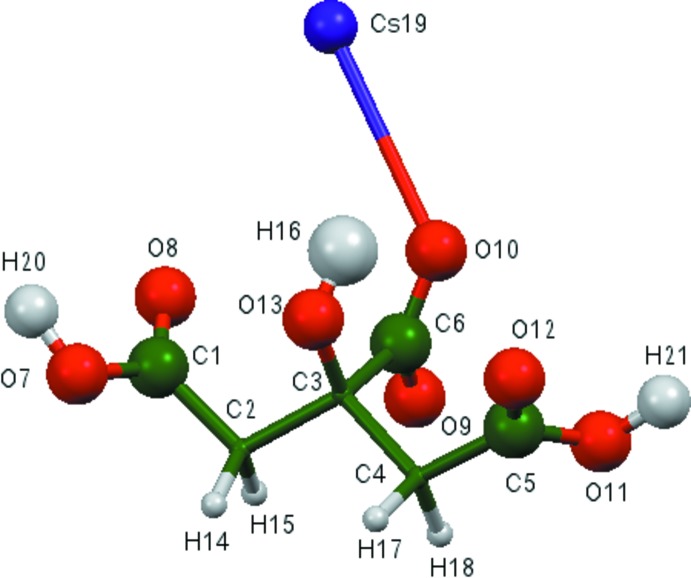
The asymmetric unit, with the atom numbering. The atoms are represented by 50% probability spheroids.

**Figure 2:**
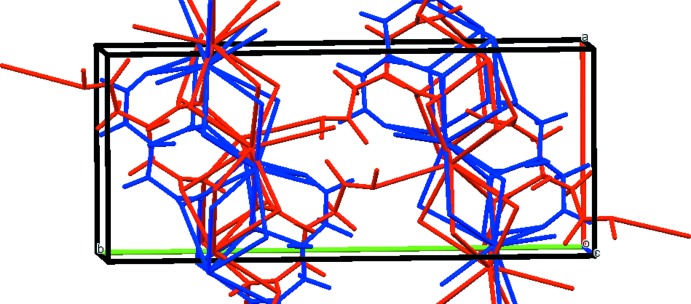
Comparison of the refined and optimized structures of caesium di­hydrogen citrate. The refined structure is in red, and the DFT-optimized structure is in blue.

The asymmetric unit of the title compound is shown in Fig. 1 ▸. The root-mean-square deviation of the non-hydrogen atoms in the Rietveld-refined and DFT-optimized structures is 0.387 Å (Fig. 2 ▸). This agreement is at the upper end of the range of correct structures as discussed by van de Streek & Neumann (2014 ▸). Re-starting the Rietveld refinement from the DFT-optimized structure led to higher residuals (*R_wp_* = 0.1287 and χ^2^ = 26.43). Accurate determination of the positions of C and O atoms in the presence of the heavy Cs atoms using X-ray powder data might be expected to be difficult. This discussion uses the DFT-optimized structure. Most of the bond lengths, bond angles, and torsion angles fall within the normal ranges indicated by a *Mercury* Mogul geometry check (Macrae *et al.*, 2008 ▸), but the torsion angles involving the central carboxyl­ate and hydroxyl group are flagged as unusual; the central portion of the mol­ecule is less-planar than usual. In the refined structure, the O8—C1 and O10—C6 bonds, as well as the C3—C2—C1 angle, were flagged as unusual. The citrate anion occurs in the *trans,trans* conformation, which is one of the two low-energy conformations of an isolated citrate. The central carboxyl­ate O10 and the terminal carboxyl­ate O12 atoms chelate to the Cs^+^cation. The Mulliken overlap populations and atomic charges indicate that the metal-oxygen bonding is ionic.

The Bravais–Friedel–Donnay–Harker (Bravais, 1866 ▸; Friedel, 1907 ▸; Donnay & Harker, 1937 ▸) morphology suggests that we might expect a platy morphology for cesium di­hydrogen citrate, with {020} as the principal faces. A 4th-order spherical harmonic texture model was included in the refinement. The texture index was 1.183, indicating that preferred orientation was significant for this rotated flat-plate specimen.

## Supra­molecular features   

**Figure 3:**
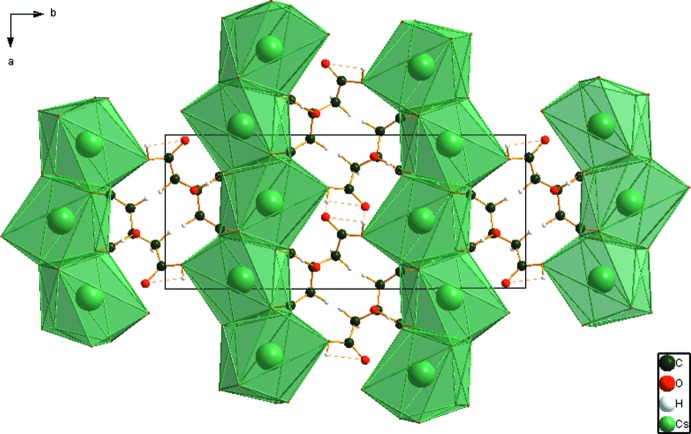
Crystal structure of CsH_2_C_6_H_5_O_7_, viewed down the *c*-axis.

The nine-coordinate Cs^+^ cation (bond-valence sum 0.96) share edges to form chains along the *a* axis (Fig. 3 ▸). These chains are linked by corners along the *c* axis. The O7—H20⋯O8 hydrogen bonds (Table 1 ▸) form a helical chain along the *c* axis, and the O11—H21⋯O10 hydrogen bonds are discrete. The Mulliken overlap populations in these hydrogen bonds are 0.064 and 0.095 *e*, respectively. By the correlation in Rammohan & Kaduk (2017*a* ▸), these hydrogen bonds contribute 13.8 and 16.8 kcal mol^−1^ to the crystal energy. The hy­droxy group O13—H16 acts as a donor in two hydrogen bonds. The one to O10 is intra­molecular, with a graph-set symbol *S*(5). The one to O9 is inter­molecular, with a graph set symbol *S*(7). These hydrogen bonds are weaker, contributing 11.2 and 9.1 kcal mol^−1^ to the crystal energy.

## Database survey   

Details of the comprehensive literature search for citrate structures are presented in Rammohan & Kaduk (2017*a* ▸). A reduced-cell search of the cell of cesium di­hydrogen citrate in the Cambridge Structural Database (Groom *et al.*, 2016 ▸) (increasing the default tolerance from 1.5 to 2.0%) yielded 60 hits, but combining the cell search with the elements C, H, Cs, and O only yielded no hits.

## Synthesis and crystallization   

H_3_C_6_H_5_O_7_(H_2_O) (2.0766 g, 10.0 mmol) was dissolved in 10 ml deionized water. Cs_2_CO_3_ (1.6508 g, 5.0 mmol, Sigma–Aldrich) was added to the citric acid solution slowly with stirring. A white precipitate formed in about two minutes, and the colourless solution was evaporated to dryness at ambient conditions.

## Refinement details   

**Figure 4:**
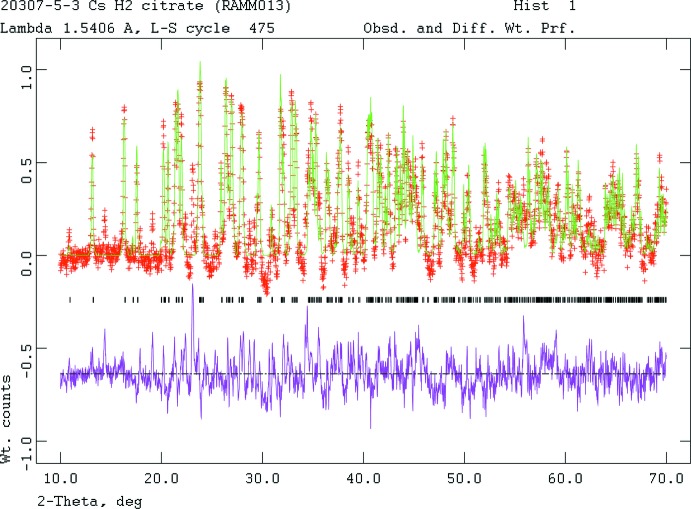
Rietveld plot for the refinement of CsH_2_C_6_H_5_O_7_. The vertical scale is not the raw counts but the counts multiplied by the least squares weights. This plot emphasizes the fit of the weaker peaks. The red crosses represent the observed data points, and the green line is the calculated pattern. The magenta curve is the difference pattern, plotted at the same scale as the other patterns. The row of black tick marks indicates the reflection positions.

Crystal data, data collection and structure refinement details are summarized in Table 2 ▸. The powder pattern (Fig. 4 ▸) was indexed using *DICVOL06* (Louer & Boultif, 2007 ▸) [M/F(18) = 64/117] on a primitive ortho­rhom­bic unit cell having *a* = 8.7362 (2), *b* = 20.5351 (2), *c* = 5.1682 (5) Å, *V* = 927.17 (9) Å^3^, and *Z* = 4. The peak list from a Le Bail fit in *GSAS* was imported into Endeavour 1.7b (Putz *et al.*, 1999 ▸), and used for structure solution. The successful solution used a citrate, a Cs atom, and two oxygen atoms from water mol­ecules. Initial Rietveld refinements moved the oxygens close to the Cs site, so they were deleted from the refinement.

Pseudo-Voigt profile coefficients were as parameterized in Thompson *et al.* (1987 ▸) with profile coefficients for Simpson’s rule integration of the pseudo-Voigt function according to Howard (1982 ▸). The asymmetry correction of Finger *et al.* (1994 ▸) was applied, and microstrain broadening by Stephens (1999 ▸). The structure was refined by the Rietveld method using *GSAS/EXPGUI* (Larson & Von Dreele, 2004 ▸; Toby, 2001 ▸).

All C—C and C—O bond lengths were restrained. The C—C bonds were restrained at 1.54 (1) Å, and the C3—O13 bond at 1.42 (2) Å. The C—O bonds in the carboxyl­ate groups were restrained at 1.26 (2) Å. All angles were also restrained; the restraints were 109 (3)° for the angles around tetra­hedral carbon atoms, and 120 (3)° for the angles in the planar carboxyl­ate groups. The restraints contributed 3.0% to the final χ^2^. The hydrogen atoms were included at fixed positions, which were recalculated during the course of the refinement using *Materials Studio* (Dassault Systèmes, 2014 ▸).

## DFT calculations   

A density functional geometry optimization (fixed experimental unit cell) was carried out using *CRYSTAL09* (Dovesi *et al.*, 2005 ▸). The basis sets for the C, H, and O atoms were those of Gatti *et al.* (1994 ▸), and the basis set for Cs was that of Prencipe (1990 ▸). The calculation used 8 *k*-points and the B3LYP functional, and took about 59 h on a 2.4 GHz PC. *U*_iso_ were assigned to the optimized fractional coordinates based on the *U*_iso_ from the refined structure.

## Supplementary Material

Crystal structure: contains datablock(s) RAMM013_publ, ramm013_DFT. DOI: 10.1107/S2056989017000135/vn2124sup1.cif

CCDC references: 1525625, 1525624

Additional supporting information:  crystallographic information; 3D view; checkCIF report

## Figures and Tables

**Table 1:** Hydrogen-bond geometry (Å, °)

*D*—H⋯*A*	*D*—H	H⋯*A*	*D*⋯*A*	*D*—H⋯*A*
O11—H21⋯O10^i^	1.028	1.575	2.600	174.4
O7—H20⋯O8^ii^	0.996	1.674	2.637	161.7
O13—H16⋯O9^iii^	0.979	1.985	2.865	148.4
O13—H16⋯O10	0.979	2.149	2.691	113.3

Symmetry codes: (i) 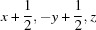; (ii) 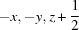; (iii) 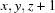.

**Table 2:** Experimental details

	Powder data
Crystal data	
Chemical formula	Cs^+^·H_2_C_6_H_5_O_7_^−^
*M*_r_	323.97
Crystal system, space group	Orthorhombic, *P* *n* *a*2_1_
Temperature (K)	300
*a*, *b*, *c* (Å)	8.7362 (2), 20.53510 (16), 5.1682 (5)
*V* (Å^3^)	927.17 (9)
*Z*	4
Radiation type	*K*α_1_, *K*α_2_, λ = 1.540629, 1.544451 Å
Specimen shape, size (mm)	Flat sheet, 24 × 24

Data collection	
Diffractometer	Bruker D2 Phaser
Specimen mounting	Standard holder
Data collection mode	Reflection
Scan method	Step
2θ values (°)	2θ_min_ = 5.042 2θ_max_ = 70.050 2θ_step_ = 0.020

Refinement	
*R* factors and goodness of fit	*R*_p_ = 0.068, *R*_wp_ = 0.089, *R*_exp_ = 0.026, *R*(*F* ^2^) = 0.171, χ^2^ = 11.765
No. of parameters	57
No. of restraints	29
H-atom treatment	Only H-atom displacement parameters refined

The same symmetry and lattice parameters were used for the DFT calculation. Computer programs: *DIFFRAC.Measurement* (Bruker, 2009 ▸), *GSAS* (Larson & Von Dreele, 2004 ▸), *DIAMOND* (Crystal Impact, 2015 ▸) and *publCIF* (Westrip, 2010 ▸).

## supplementary crystallographic information

### Crystal data

**Table:**

CsH_2_C_6_H_5_O_7_	*c* = 5.1682 Å
*M**_r_* = 323.97	*V* = 927.17 Å^3^
Orthorhombic, *P**n**a*2_1_	*Z* = 4
Hall symbol: P 2c -2n	*D*_x_ = 2.321 Mg m^−^^3^
*a* = 8.7362 Å	Cu *K*α radiation, λ = 1.5418 Å
*b* = 20.5351 Å	*T* = 300 K

### Data collection

**Table:**

Density functional calculation	*k* = →
*h* = →	*l* = →

### Fractional atomic coordinates and isotropic or equivalent isotropic displacement parameters (Å^2^)

**Table:**

	*x*	*y*	*z*	*U*_iso_*/*U*_eq_	
C1	0.14729	0.01007	0.32268	0.06500*	
C2	0.28682	0.02722	0.16639	0.00900*	
C3	0.35878	0.08945	0.28492	0.00900*	
C4	0.52514	0.09664	0.18015	0.00900*	
C5	0.60947	0.15212	0.30828	0.06500*	
C6	0.26991	0.15158	0.20135	0.06500*	
O7	0.15730	−0.04600	0.44742	0.06500*	
O8	0.03401	0.04586	0.34061	0.06500*	
O9	0.23818	0.15901	−0.03279	0.06500*	
O10	0.24301	0.19347	0.37889	0.06500*	
O11	0.62642	0.20442	0.15627	0.06500*	
O12	0.65699	0.15090	0.53096	0.06500*	
O13	0.36027	0.08089	0.55685	0.06500*	
H14	0.36802	−0.01307	0.17323	0.01200*	
H15	0.25479	0.03604	−0.03477	0.01200*	
H16	0.31496	0.11870	0.64320	0.08500*	
H17	0.58685	0.05170	0.22176	0.01200*	
H18	0.52243	0.10375	−0.02890	0.01200*	
Cs19	−0.05606	0.21080	0.74122	0.05030*	
H20	0.06940	−0.05097	0.56860	0.05000*	
H21	0.67528	0.24300	0.25240	0.05000*	

### Bond lengths (Å)

**Table:**

C1—C2	1.504	C4—H17	1.090
C1—O7	1.322	C4—H18	1.091
C1—O8	1.354	C5—O11	1.339
C2—C3	1.550	C5—O12	1.224
C2—H14	1.090	C6—O9	1.251
C2—H15	1.092	C6—O10	1.279
C3—C4	1.558	O7—H20^i^	0.996
C3—C6	1.555	O11—H21	1.028
C3—O13	1.416	O13—H16	0.979
C4—C5	1.510	H20—O7^ii^	0.996

Symmetry codes: (i) *x*−1/2, −*y*+1/2, *z*; (ii) *x*+1/2, −*y*+1/2, *z*.

### Hydrogen-bond geometry (Å, º)

**Table:**

*D*—H···*A*	*D*—H	H···*A*	*D*···*A*	*D*—H···*A*
O11—H21···O10^ii^	1.028	1.575	2.600	174.4
O7—H20···O8^iii^	0.996	1.674	2.637	161.7
O13—H16···O9^iv^	0.979	1.985	2.865	148.4
O13—H16···O10	0.979	2.149	2.691	113.3

Symmetry codes: (ii) *x*+1/2, −*y*+1/2, *z*; (iii) −*x*, −*y*, *z*+1/2; (iv) *x*, *y*, *z*+1.
